# Lipid profile dataset of optogenetics induced optic nerve regeneration

**DOI:** 10.1016/j.dib.2020.106001

**Published:** 2020-07-05

**Authors:** Jennifer Arcuri, Yuan Liu, Richard K. Lee, Sanjoy K. Bhattacharya

**Affiliations:** a Bascom Palmer Eye Institute, Miller School of Medicine at University of Miami, Miami, FL 33136, United States; b Miami Integrative Metabolomics Research Center, Miami, FL 33136, United States; c Molecular Cellular Pharmacology Graduate Program, University of Miami, Miami, FL 33136, United States

**Keywords:** Optic nerve crush, Optogenetics, Lipids, Regeneration

## Abstract

The optic nerve transfers visual information from the retina to the brain through the axons of retinal ganglion cells (RGCs). In adult mammals, optic nerve injuries and progressive degenerative diseases lead to the irreversible loss of RGCs, resulting in vision loss and blindness. Optogenetic models have proved useful in manipulating the growth of RGCs through expression and stimulation of channelrhodopsins (Chr2) in RGCs using the RGC-specific thy-1 promoter. Using transgenic Chr2 mouse (Thy1-ChR2-EYFP) as a model of regeneration, we profile the lipid changes which occur after traumatic optic nerve crush, light stimulation and forced RGC axonal growth. Thy1-ChR2-EYFP and control (C57BL/6) mice were divided in four groups each – 1) no crush and no stimulation, 2) no crush with stimulation, 3) crush and without stimulation, and 4) crush with stimulation. After euthanasia, the optic nerves were collected for lipidomic analysis. The Bligh and Dyer method was used for lipid extraction, followed by mass spectrometry lipid profiling with a Q-Exactive Orbitrap Liquid Chromatography-Mass Spectrometer (LC MS-MS). The raw scans were analysed with LipidSearch 4.1.3 and the statistical analysis was conducted through Metaboanalyst 4.0. This data is available at Metabolomics Workbench, study ID ST001381: [https://www.metabolomicsworkbench.org/data/DRCCMetadata.php?Mode=Study&StudyID=ST001381&StudyType=MS&ResultType=5].

Specifications Table

**Table:** 

Subject	Ophthalmology
Specific subject area	Lipids
Type of data	Chart Graph Figure Chromatograms Spectra
How data were acquired	Liquid Chromatography Q-Exactive Orbitrap Mass Spectrometry
Data format	Raw analysed Filtered
Parameters for data collection	Optic Nerves, Optogenetics, Light stimulation
Description of data collection	Optic nerves from transgenic channelrhodopsin mice (Thy1-ChR2-EYFP mice) and C57BL/6 J mice were collected. Thy1-ChR2-EYFP mice and controls were divided in four groups each (*n* = 6) – 1) no crush and no stimulation, 2) no crush and stimulation, 3) crush and no stimulation, and 4) crush and stimulation. Optic nerves were collected, and lipid extraction was performed with the Bligh and Dyer method. The samples were analysed untargeted for lipids with Liquid Chromatography Q-Exactive Orbitrap Mass Spectrometry.
Data source location	Bascom Palmer Eye Institute, Miller School of Medicine at University of Miami, Miami, FL 33,136, USA
Data accessibility	Study ID ST001381 at Metabolomics Workbench Repository: https://www.metabolomicsworkbench.org/data/DRCCMetadata.php?Mode=Study&StudyID=ST001381&StudyType=MS&ResultType=5

## Value of the Data

• The data provides insights into the lipid changes that occur after traumatic optic nerve injury and axonal regeneration in two mice models - Thy1-ChR2-EYFP mice and C57BL/6 J control mice.
• This data is beneficial for the study of optic nerve regeneration.
• The data can be used to further study and understand the biology of optic nerve regeneration and the lipid changes involved.
• This data can be used create lipid spectral libraries for targeted lipidomic experiments of the two mouse models investigated.

## 1 Data description

**Fig. 1:**
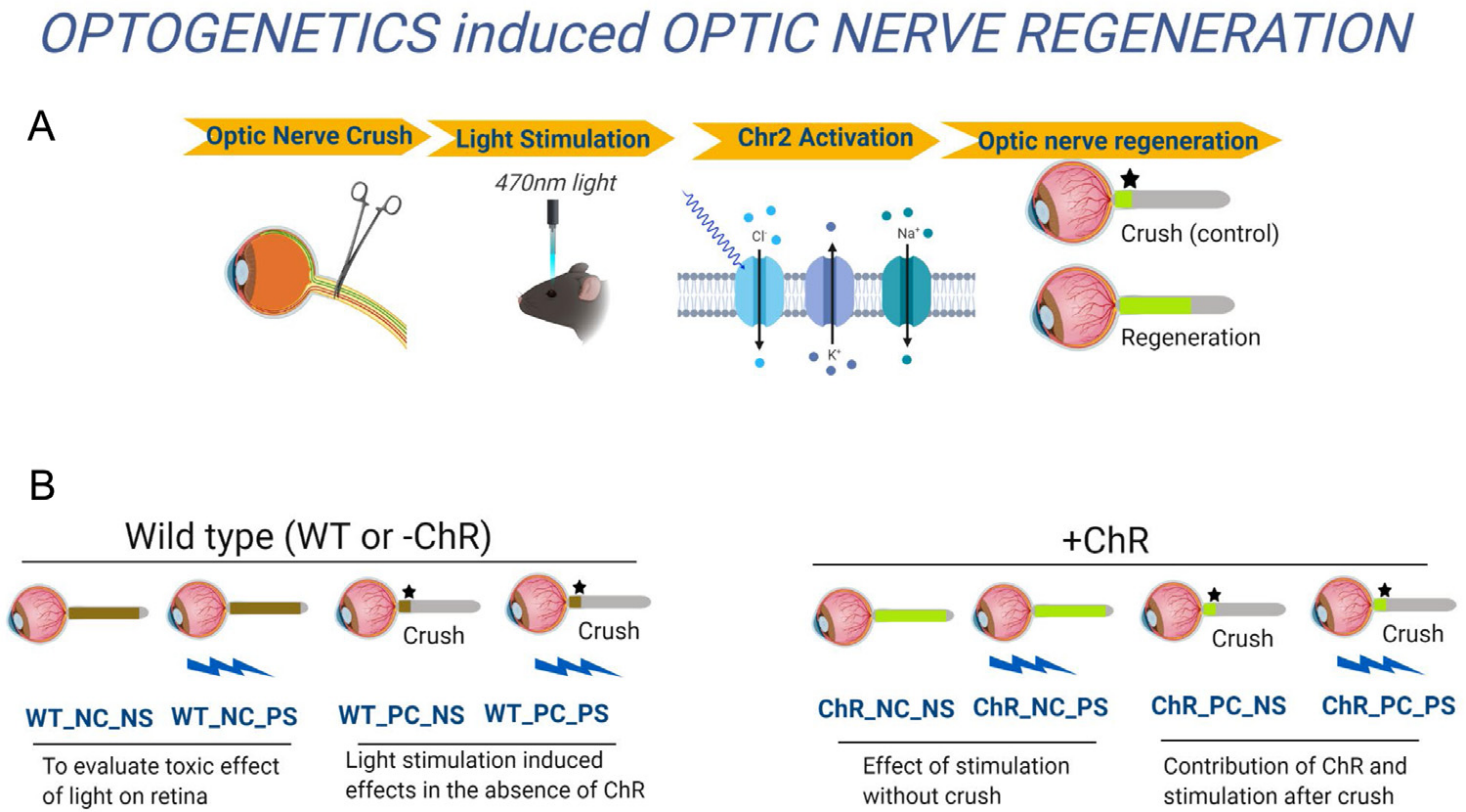
Lipid Profiling of optogenetics induced optic nerve regeneration. A. Schematic representation of the optogenetics mouse model. B. The dataset consists of the following experimental groups: Wild type (WT or Channelrodopsin(ChR) NC_NS (without optic nerve (ON) crush and no light stimulation), NC_PS (without ON crush and given light stimulation), PC_NS (with ON crush and no light stimulation) and PC_PS (with optic nerve crush and light stimulation).

**Fig. 2:**
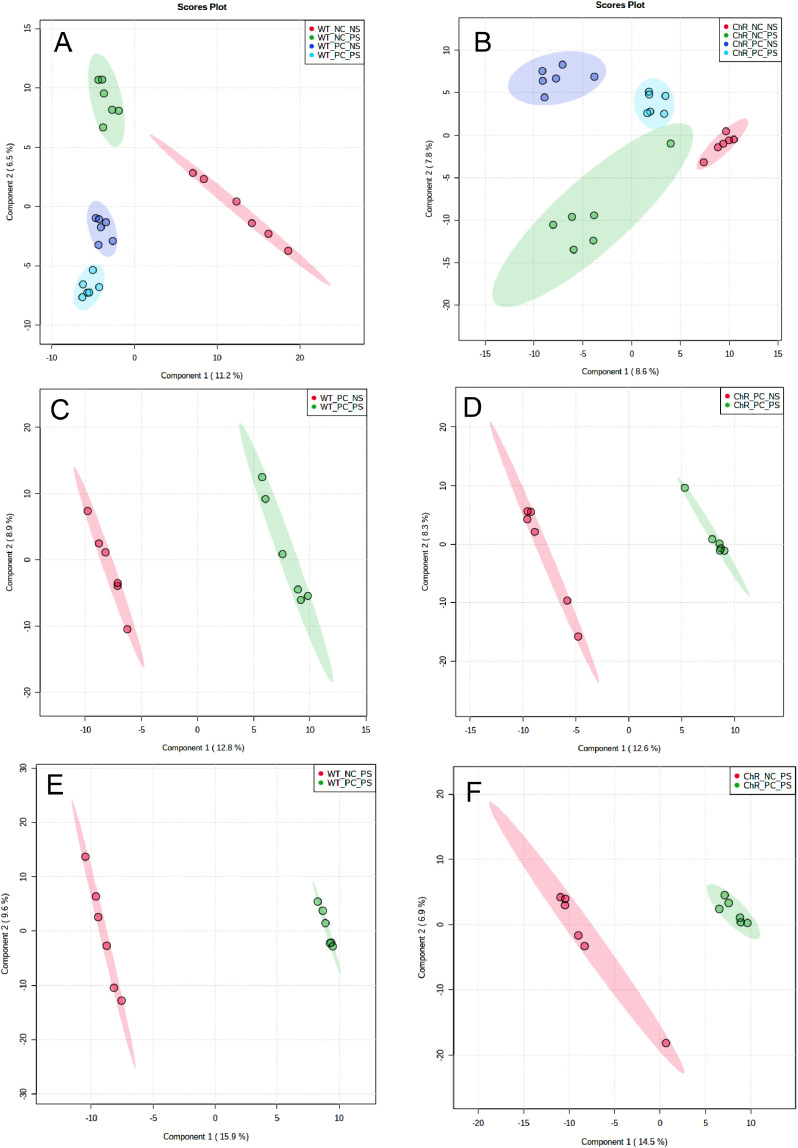
Partial least square discriminant analyses of the lipid profiling data. A., B. Wild type and recombinant channel rhodopsin (Chr) all groups: non crushed (NC), Crushed (PC), light stimulated (PS) and non stimulated (NS) groups. C-F. Wild type and ChrNS, PS groups of NC or PC as indicated. Data from Supplemental Table Sxxwas used for analysis. Samples are plotted in 2 dimensions using their projection onto the first 2 principal components (within parenthesis% of total variance explained).

**Fig. 3:**
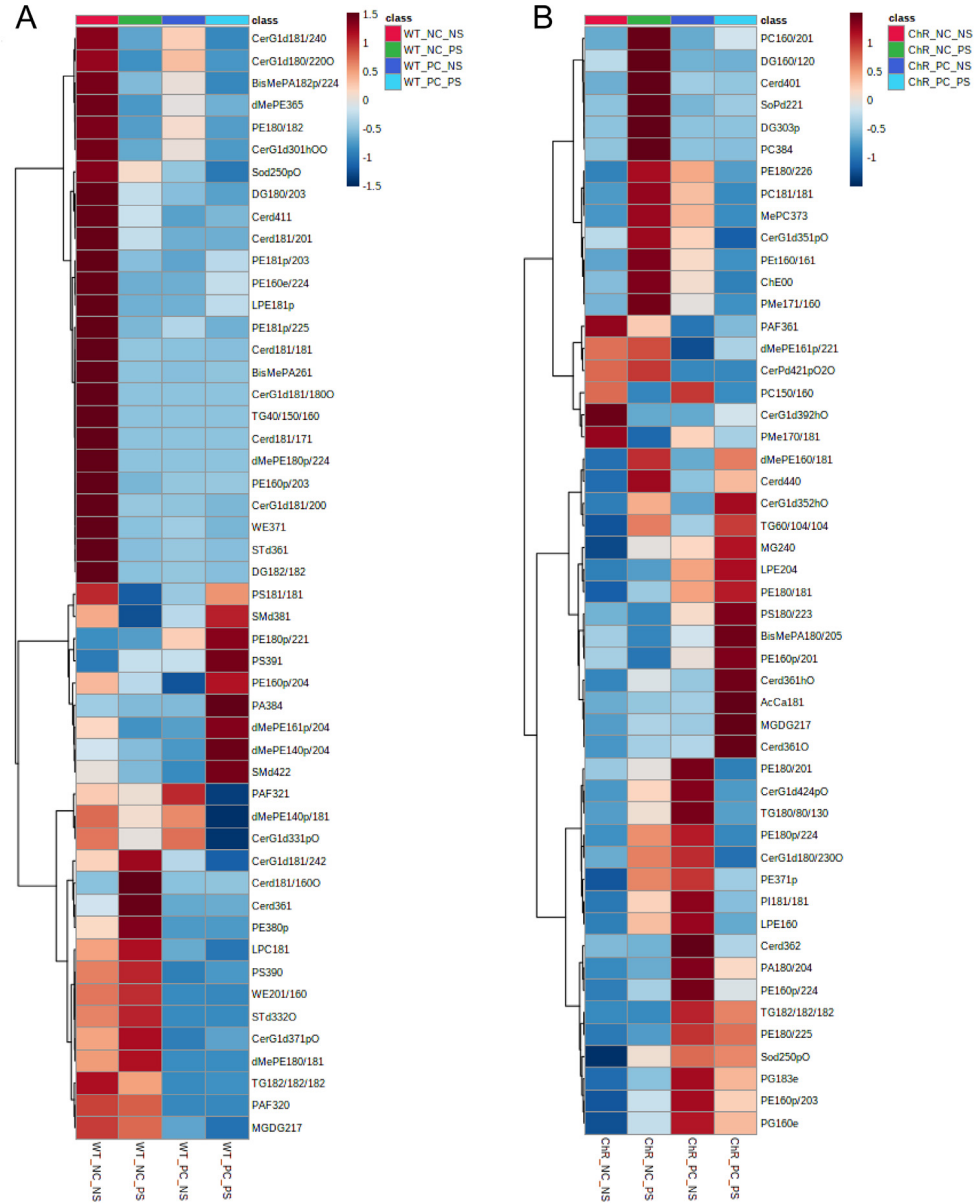
Heatmapof the lipid abundance changes in the optic nerve 15 days post-crush. A. Wild type and B. Channel rhodopsin expressing transgenic animals. Significant species are presented (FDR-adjusted p-value 0.05; one-way ANOVA). Ward clustering algorithm, Euclidean distance measure, autoscale. NC = non optic nerve crushed, PC = Optic nerve crushed, NS = Non lightstimulated and PS = Light stimulated groups.

Mass-Spectrometric lipidomics analysis was performed on optic nerves from the transgenic channelrhodopsin 2 mouse (Thy1-ChR2-EYFP) as a model for stimulated axonal regeneration and from control C57BL/6 J mouse after optic nerve crush and light stimulation to activate cation channels through ChR2 to stimulate RGCs. Optogenetics employs genetic and optical methods for the precise stimulation of specific cell types which express ChR2 [1]. The Thy1-ChR2-EYFP mouse expresses channelrhodopsin, under the thy-1 RGC specific promoter, in retinal ganglion cells (RGCs) which are specifically targeted for stimulation by ChR2 cation channel activating blue light [2]. The experimental groups (Fig. 1) evaluated toxic effects of light on the retina (WT_NC_NS and WT_NC_PS), light stimulation induced effects in the absence of channel rhodopsin (WT_PC_NS and WT_PC_PS), effects of stimulation without crush (ChR_NC_NS and ChR_NC_PS) and contribution of ChR and stimulation after crush (ChrR_PC_NS and ChR_PC_PS). The raw scans from a Q-Exactive Orbitrap Mass Spectrometer were analysed with Lipidsearch 4.1.3. All raw data were uploaded and processed with Lipidsearch 4.1.3. The aligned data were normalized and exported for bioinformatics analysis. Partial least square discriminant analyses of the lipid profiling data (Fig. 2) was obtained with Metaboanalyst 4.0, as well at the lipid species heatmap (Fig. 3).

## 2 Experimental design, materials, and methods

### 2.1 Animals

Mice were cared in accordance with the National Research Council's Guide for the Care and Use of Laboratory Animals and the Association for Research in Vision and Ophthalmology (ARVO) Statement for the Use of Animals in Ophthalmic and Vision Research. All procedures involving animals were approved by the Institutional Animal Care and Use Committee (IACUC) at the University of Miami. Thy1-Chr2-eYFP and C57BL6/6 J mice were purchased from Jackson Laboratory (stock numbers 007,615 and 000,664), Bar harbor, Maine, USA.

For the optic nerve crush, Thy1-Chr2-eYFP and C57BL/6 J mice at age two months were anesthetized using a ketamine/xylazine cocktail injected intraperitoneally and eyes were additionally topically anesthetized using 0.5% proparacaine hydrochloride. A surgical peritomy was made behind and above the eyeball and the eye muscles were gently retracted to expose the optic nerve. Dumont #5 forceps (Fine Science Tools, Foster City, CA, USA) were used to crush the optic nerve approximately 0.5–1 mm behind the globe without damaging retinal vessels or affecting the blood supply as described previously [3]. Mice of either sex were randomly assigned into optogenetic stimulation and control groups. To deliver pulsed blue light to optically sensitive RGCs, 20 blue light-emitting diodes (LEDs, emission peak at 465 nm, Cree, Durham, NC, USA) were soldered on metal core printed circuit board (PCB)(LEDsupply, Randolph, VT, USA). The frequency was controlled by a digital cycle timer switch that can be programmed to have different frequency settings (Inkbird, Shenzhen, China). The blue light was set to keep off for 59.5 s and on for 0.5 s. The mice in stimulated environment received blue light stimulation for consecutive two or four weeks after optic nerve crush. The mice in control environment were kept in normal 12 h light/dark cycle for the same time as those in the stimulated environment.After euthanasia, the optic nerves were dissected for analysis.

Twenty blue light LEDs were fixed on a special ion cage and the mice housing cage was placed in the ion cage. The output power of each LED is 10 mW. We observed axon regeneration in the Chr2 mice receiving blue light stimulation after optic nerve crush. To verify this axon regeneration is caused by the activation of Chr2 protein, we conducted two sets of control experiments. First, we put Chr2 mice in the normal environment after crush and we didn't observe any axon regeneration. Second, we put mice that have same genetic background but do not express Chr2 protein in the blue light stimulation environment after crush, we also did not observe any axon regeneration in this set of mice. Our studies have found that, the activation of Chr2 protein by blue light is required for the optic nerve regeneration after injury.

### 2.2 Lipid extraction

A modification of the Bligh and Dyer method was used for lipid extraction with LC-MS grade solvents. Dissected optic nerves were stored at −80 °C and treated with 6 mL of methanol and 3 mL of chloroform per sample. The samples were vortexed and sonicated in ultrasonic bath for 2 min and incubated overnight at 48 °C. This was followed by addition of 2 mL of water and 1.5 mL of chloroform. For protein and lipid separation, the samples were vortexed again fro 2 min and centrifuged at 3000 RCF for 15 min. The lower phase was collected and dried completely in a centrifugal vacuum concentrator.

### 2.3 High performance liquid chromatography and mass spectrometry

Lower phase samples containing the lipids were resuspended in 60 µl of chloroform: methanol 1:1 (v/v) followed by a 20 min ultrasonic water bath and 2 min vortex. Samples were anlaysed by using liquid chromatography electrospray tandem mass spectrometry (LC-MS/ MS). The instruments used were the Accela HPLC system and an orbitrap mass spectrometer (Q-Exactive, Thermo Scientific, Waltham, MA). An Acclaim 120 C18 3 µm column (Thermo Scientific) was run with methanol: water 60:40 (v/v) and 10 mM ammonium acetate and methanol chloroform 60:40 (v/v) with 10 mM ammonium acetate, as solvent A and B, respectively. The heated electrospray ionization source (HESI) was used with the following settings: spray voltage 4415 V, vaporization temperature 275 °C, auxilliary gas flow 15 arbitrary units.

### 2.4 Raw scan processing and bioinformatics

Lipid identification was performed using LipidSearch 4.1 software with a parent and product *m/z* tolerance of 5 ppm. All classes target classes were selected and all adduct ions except (CH_3_CH_2_)_3_NH+ and (CH_3_)_2_NH_2_. Sample alignement was performed for negative and positive ions modes. The exported file was used for analysis in Metaboanalyst 4.0, where data was scaled using the autoscaling feature. A PCA was generated (Fig. 2) for different sample comparisons as well as two heat maps for wild type and channelrhodopsin groups.

## Declaration of Competing Interest

The authors declare that they have no known competing financial interests or personal relationships which have, or could be perceived to have, influenced the work reported in this article.

## Appendix Supplementary materials

Image, application 1
